# STK31 as novel biomarker of metastatic potential and tumorigenicity of colorectal cancer

**DOI:** 10.18632/oncotarget.15396

**Published:** 2017-02-16

**Authors:** Lan Zhong, Jing Liu, Yedong Hu, Wei Wang, Fei Xu, Wen Xu, Junyi Han, Ewelina Biskup

**Affiliations:** ^1^ Department of Gastroenterology, East Hospital of Tongji University School of Medicine, Shanghai, China; ^2^ State Key Laboratory of Genetic Engineering, Institute of Genetics, School of Life Sciences, Fudan University, Shanghai, China; ^3^ Department of General Surgery, East Hospital of Tongji University School of Medicine, Shanghai, China; ^4^ Department of Internal Medicine, University Hospital of Basel, Switzerland

**Keywords:** colorectal cancer, metastasis, serine–threonine kinase 31 (STK31), cancer testis antigen

## Abstract

**Purpose:**

Colorectal cancer (CRC) is the fifth most common cause of cancer deaths in China and fourth worldwide. Metastatic dissemination of primary tumors is considered main cause for CRC related mortality. The serine–threonine kinase 31 (STK31) gene is a novel cancer testis (CT) antigen. It was found significantly highly expressed in gastrointestinal cancers. In our study we aimed to analyze the correlation between STK31 expression patterns and metastasization, tumor stage and grade in CRC patients.

**RESULTS:**

Relative STK31 expression level was significantly higher in patients with lymph node metastasis. STK31 expression levels in primary tumorous tissues of metastatic patients were significantly higher than in ANCTs and in lymph nodes samples, both at the RNA level and the protein level.

**Materials and Methods:**

Surgical specimens of cancerous tissues, paired with adjacent noncancerous tissues, and lymph nodes from 44 CRC cases with different clinicopathological features were collected. Expression of STK31 was detected and measured by immunohistochemistry and quantitative real-time polymerase chain reaction (QRT-PCR).

**Conclusions:**

Our data suggest that STK31 might be a potential biomarker in detecting, monitoring and predicting the metastatic risk of colorectal cancer.

## INTRODUCTION

Modern diet and lifestyle contributed greatly towards a raise of colorectal cancer (CRC) occurrence, which became an emergent health care burden worldwide. Its global incidence continues to increase, especially in developed, welfare countries. GLOBOCAN 2012 (International Agency for Research on Cancer, http://globocan.iarc.fr/Default.aspx) estimated over 373631 CRC deaths, accounting for 8% of all cancer deaths, positioning CRC as the fifth most common cause of cancer deaths in China and the fourth worldwide. The golden clinical standard for colorectal cancer prophylaxis and diagnostics is colonoscopy. Because of its invasive nature and insidious, often silent progression, the majority of CRC patients are diagnosed only at an advanced stages [1], where therapeutic regimens are limited and prognosis poorly. If detected early enough, the tumor can be treated with excellent results, providing the patient with a good long-term prognosis and long recurrence-free interval. Thus, systematic primary prophylaxis, early diagnosis and rapid intervention are critical for reducing CRC mortality [2, 3].

Despite a growing number of genes identified and investigated as biomarkers in CRC, such as SPAG9 or PTCH1 [1, 4], none is routinely used for clinical detection and anticancer treatment. Therefore, it is vital to explore biomarkers more profoundly and design simple methods to implement them into prophylactic the therapeutic, as well as monitoring and prognostic approaches. Especially useful would be the application of genetic biomarkers to detect and predict CRC metastasis. Identification of specific measurable molecules that are associated with tumor aggressiveness would be of great clinical significance, providing options for assessing early spread of CRC.

The serine–threonine kinase 31 (STK31) gene was initially identified through cDNA subtraction as a testis-specific protein kinase gene expressed in mouse spermatogonia [5, 6]. Immunolocalization of equine STK31 revealed a specific localization in the equatorial region of post-meiotic spermatocytes, elongating spermatids, and ejaculated spermatozoa [7]. Human STK31 gene, mapped to chromosome 7p15.3, has two splice variants reported to date, which encode a putative 1,019 a.a. protein (isoform a; accession #NP113602) and a putative 996 a.a. protein (isoform b; accession #NP116562). Although the biological function of STK31 remains to be determined, its highly conserved domain structure between vertebrate species, including a tudor domain, a SbcC domain as well as the putative serine–threonine kinase domain [7], indicates that it might be involved in DNA repairing/repackaging and chromatin remodeling during spermiogenesis, sperm maturation, and sperm function [8–11]. Recently, STK31 has been described as a novel cancer testis (CT) antigen, highly expressed in GI cancer cells (colorectal, gastric and esophageal cancer), while restricted to testis and fetal brain in normal tissues [12]. Mori et al [12] reported that STK31-derived peptide was able to trigger specific cytotoxic T-lymphocytes (CTLs) and to induce CTLs cell lysis in a HLA-A*0201–restricted manner or by peptide-loading. STK31 is thus a potential diagnostic biomarker for CRC, a good candidate for targeted therapy and monitoring, as well as an assumptive predictive and prognostic factor for early stage and metastatic CRC.

In the present study, we aimed to conduct an accurate quantification of STK31 in order to investigate the correlation of its expression and CRC features. Besides of immunohistochemistry, we also carried out a real-time RT-PCR to measure mRNA expression in human CRC tissues and their adjacent non-cancerous samples. Our findings demonstrated a close relationship between STK31 expression between tumor stage and lymph node metastasis in colorectal cancer, suggesting a potential functional role in tumor progression.

## RESULTS

### Relative quantification of STK31 mRNA expression in tumors and adjacent noncancerous tissues

mRNA levels of STK31 were measured by quantitative real-time RT-PCR. Measurements were normalized using cycle threshold (CT) method. STK31 mRNA in the target tissue was related to the paired internal control and calculated as 2^−ΔCT^ (ΔCT = CT(STK31)-CT (GAPDH)). When compared, STK31 expression level in CRC tissues and that in adjacent noncancerous tissues (ANCTs) differed significantly.

### STK31 mRNA expression in relation to lymph node status

**Figure 1:**
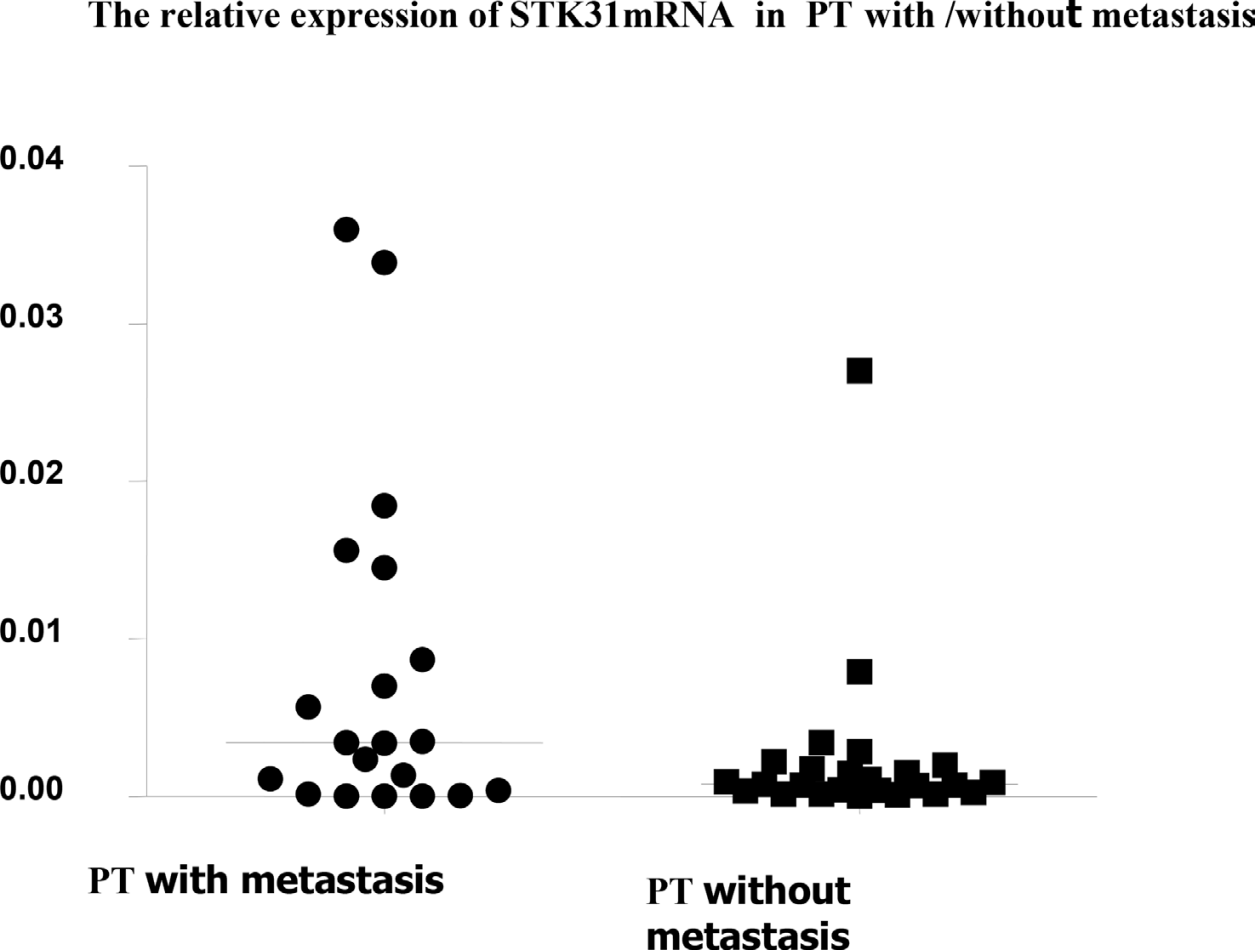
The relative STK31mRNA expression in primary tumor samples from patients with metastasis is significantly higher than in those without metastasis (0.00778 ± 0.0108 *VS* 0.00240 ± 0.0531, *P* = 0.003)

We divided the patients into two groups according to their lymph node (LN) metastatic status. Patients without metastatic lymph nodes were placed in group A and patients with metastatic lymph nodes in group B. We found a significantly higher relative STK31 expression in primary tumor samples from patients with metastasis (group B) than in those without metastasis (group A) ((0.00778 ± 0.0108 *VS* 0.00240 ± 0.0531, *,P* = 0.003, Figure 1), confirmed by stratified expression level analysis.

**Figure 2:**
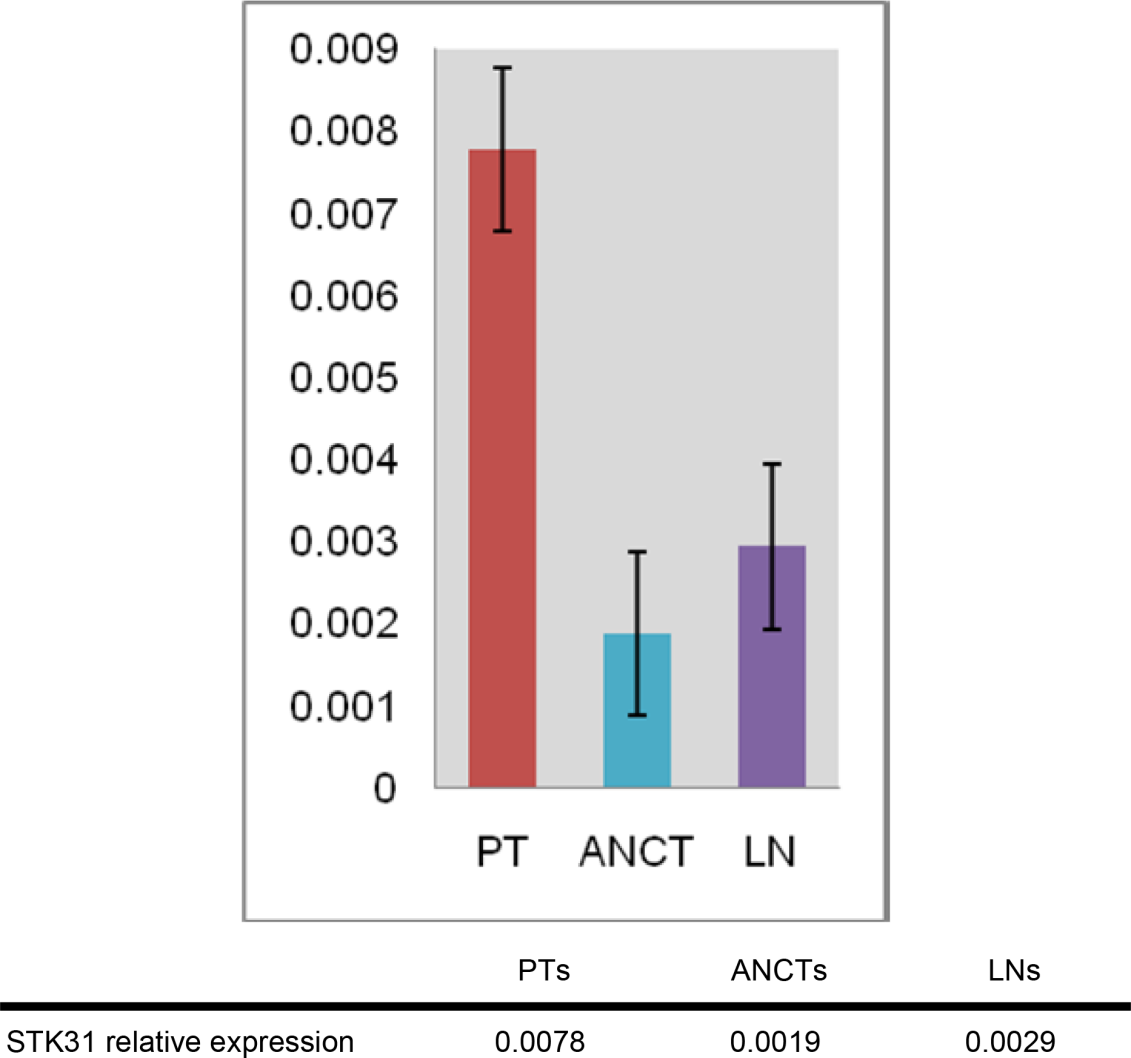
STK31 mRNA levels in tumor samples, adjacent noncancerous tissues and lymph nodes in CRC patients with metastasis, as determined by real-time RT-PCR PT: primary tumorous tissue; ANCT: adjacent noncancerous tissue; LN: lymph node.

In group A (non-LN-metastatic patients), there was no statistically significant correlation between tumor tissues and ANCTs (*P* = 0.255). In group B (metastatic group), STK31 expression levels in the primary tumorous tissues were much higher than in ANCTs and lymph nodes (Figure 2). Statistical One-way ANOVA analysis performed on group B (metastatic patients) revealed a significant difference between tumors, ANCTs and lymph nodes (ANOVA between groups F=0.021; between tumors and ANCTs *P* = 0.009, between tumors and lymph nodes *P* = 0.031).

The relationship between STK31 mRNA expression and clinico-pathological features in group B patients is summarized in Table 1. Significant correlations were seen for male sex, age of less than 60 years, well or moderately differentiated histologic grade, and serosal invasion (T3 stage). These data suggest a close relationship between STK31 expression and tumor cell migration and invasion (metastatic potential).

**Table 1:** Association between STK31 expression and clinicopathologic variables in metastatic CRC patients using One-way ANOVA analysis

clinicopathologic features	*n*	*F* value (*P* value between groups)	*P* value within groups		
			PT vs. ANCTs	PT vs. LNs	ANCT vs. LNs
**Total**					
	20	0.021*	0.009*	0.031*	0.630
**Gender**					
Male	14	0.033*	0.024*	0.023*	0.991
Female	6	0.415	0.192	0.508	0.502
**Age**					
≤ 60	14	0.043*	0.027*	0.032*	0.935
> 60	6	0.371	0.168	0.414	0.551
**Tumor site**					
Colon	8	0.099	0.076	0.052	0.853
Rectum	12	0.181	0.068	0.268	0.453
**Histologic grade**					
Well/moderately differentiated	15	0.025*	0.017*	0.019*	0.957
Poorly differentiated	5	0.520	0.263	0.585	0.551
**T stage**					
T2	3	0.428	0.211	0.495	0.526
T3	12	0.015*	0.015*	0.009*	0.824
T4	5	0.650	0.975	0.421	0.438
**Tumor stage**					
Stage I + II	12	0.093	0.033*	0.158	0.439
Stage III + IV	8	0.069	0.102	0.026*	0.498

Three groups: PT: primary tumorous tissue; ANCTs: adjacent noncancerous tissues; LNs: lymph nodes. *P < 0.05 (statistically significant).

### Relationship between STK31 mRNA expression level and further clinicopathological features

Statistical analysis of STK31 expression in groups of patients divided according to their clinicpathological parameters (and not to the LN metastatic status) showed no correlation between the STK31 mRNA levels and features, such as age, gender, tumor site, histologic grade, primary tumor invasion depth, or Duke's stage of the carcinoma (data not shown).

### Immunobiological measurement of STK31 protein expression in tumor patients with lymph node metastasis

**Figure 3:**
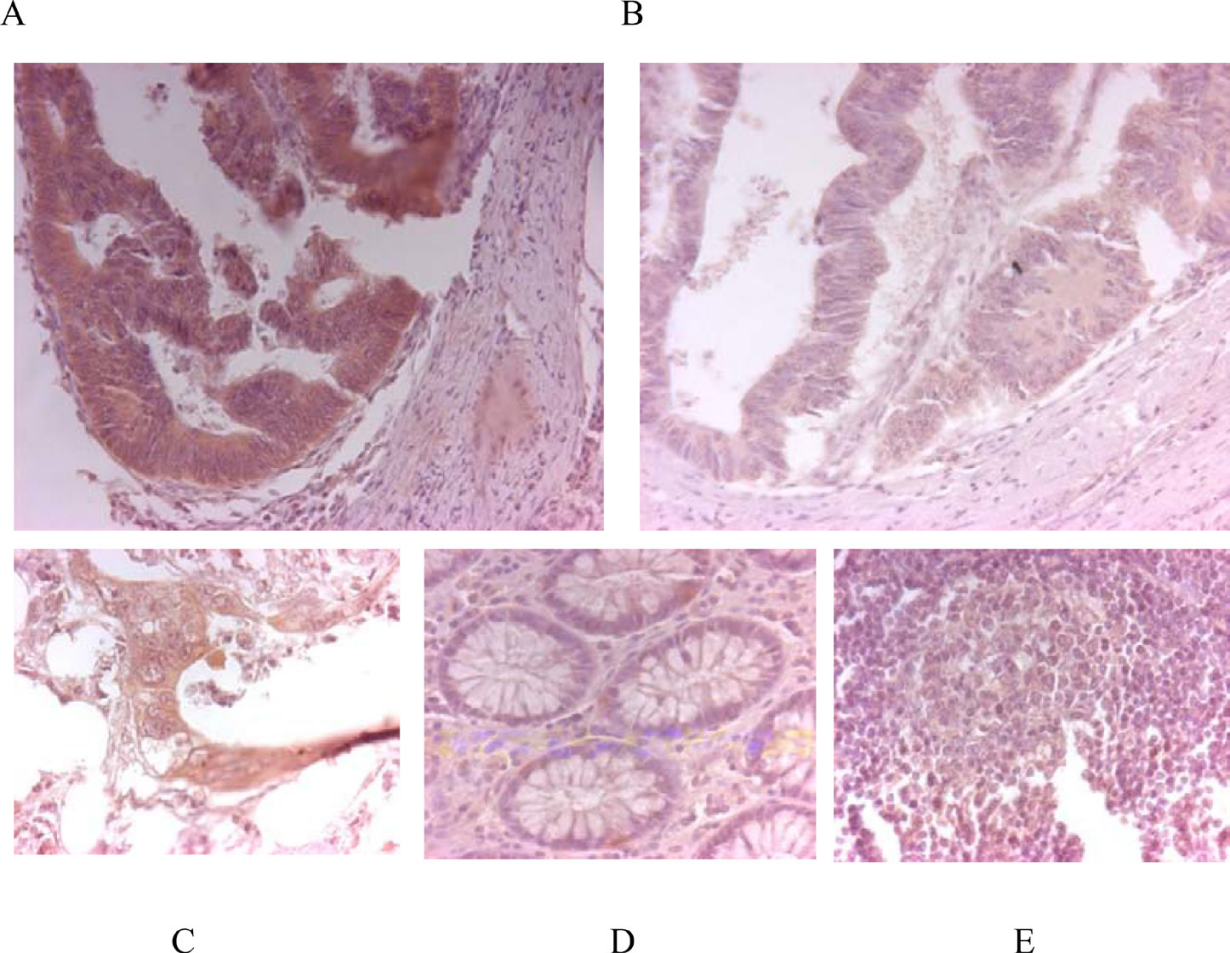
Immunohistochemistry STK31 protein expression in a primary tumor tissue of a patient with lymph node metastases (**A**) and in a patient without metastases (**B**). Primary tumor samples from a patient with lymph node metastasis: (**C**) primary tumorous tissue (strong expression), (**D**) adjacent noncancerous tissue (no expression) and (**E**) lymph node metastasis (weak expression).

STK31 expression was observed in membrane of tumor cells after immunohistological staining (Figure 3A). Consistently with the mRNA expression patterns, STK31 protein expression levels in tumor tissues of non-metastatic patients were much lower (Figure 3B). Furthermore, our immunohistochemistry staining also confirmed that the STK31 is expressed higher in primary tumorous tissues of patients with metastasis (Figure 3C) were significantly higher than in paired adjacent noncancerous tissues Figure 3D) and in lymph nodes Figure 3E).

## DISCUSSION

CRC is a malignancy with one of the highest incidences and mortalities, especially in developed countries, and represents a severe public health issue worldwide. In the past decades, CRC morbidity and mortality increased rapidly in China [4]. Despite a great improvement in diagnostics and treatment (novel chemotherapeutics, immunotherapy and molecular targeted therapy), the majority of CRC patients are diagnosed only at advanced stages and the 5-year mortality stagnates at approximately 40% [13–16]. Cell migration and invasion is a critical event in carcinogenesis and cancer progression, resulting in distant metastasis, especially to the liver [1, 17]. It has been reported that approximately 90% of CRC deaths arise from the metastatic dissemination of primary tumors, and distant metastasis has been regarded as the main cause of death in CRC patients [17–19]; still remains a challenge for oncologists to predict local recurrence, metastatic potential and outcome of primary colorectal carcinoma patients based on conventional clinical and histopathological/immunhistochemical exams [2, 20]. To date, tumor-node-metastasis (TNM) classification represents the main tool for identifying prognostic differences among patients with early-stage CRC [21]. Numerous approaches have been undertaken to characterize tumor-associated antigens. However, only few proteins have been proven to be useful biomarkers (carcinoembryonic antigen CEA, CA19.9 and CA125) [22] ). While they are applied in therapy monitoring and recurrence diagnosis, their validity in clinical screening, cancer grading and metastatic potential evaluation is not sufficient. Therefore, in order to improve treatment efficiency and efficacy, identification of new reliable, applicable and cost effective markers to detect, predict and monitor CRC metastasis in clinical practice.

We examined the clinical relevance of STK31 as a potential predictor of CRC cell migration. Our data revealed that, as a member of the CT gene family that encodes unique class of tumor-associated antigens in various malignancies, STK31 is closely related with CRC metastasis. STK31 expression in primary tumor samples of patients with lymph node metastasis was significantly higher than in those without metastasis. Moreover, STK31 expression levels in primary cancer tissues of metastatic patients were significantly higher than in ANCTs and in lymph nodes, both at mRNA and protein level. These results not only indicated that STK31 plays an important role in cell migration and invasion, but also suggested that STK31 could be a potential tumor biomarker for detecting, monitoring and predicting CRC metastatic risk, as well as individually tailor therapeutic strategies. Data of Mori et al. showed that over-expression of the STK31 gene did not contribute to progression of colorectal cancer [12]. However, this study compared STK31 expression levels in patients with and without metastasis using χ^2^ test based on an assumptive threshold to assign the data into different groups. We used Independent-Samples *t* test. Additionally, there were ethnic differences between our samples. Moreover, it has been shown that STK31 knockdown lead to a significant suppression of tumorigenicity in colon cancer cells, while STK31 over-expression promotes an undifferentiated CRC status [23]. Kuo et al [24]. proved STK31 to be a cell cycle regulated centrosome associated protein. Ectopically-expressed, STK31 increases cell migration and invasiveness of human somatic cancer cells, whereas endogenous STK31 knockdown results in microtubule assembly defects that prolong the duration of mitosis and triggers apoptosis.

Furthermore, targeting STK31 mitotic kinase can be a novel anticancer treatment [25]. STK31-derived peptide elicits specific CTLs, among others in a HLA-A*0201–restricted manner. Since HLA-A*0201 is the most commonly expressed allele and expression of CT antigens are normally restricted to immunologically privileged male germ cells, targeting STK31 in CRC patients may be a promising immunotherapeutic approach, especially in metastatic patients.

Our study provides firm evidence that STK31 is associated with metastatic CRC, and that the association can be easily measured in Real-time PCR or immunohistostaining. High STK31 expression in resected tissues could assist the leading oncologist to suggest an appropriate management for individual patients. It is striking that the expression of STK31 in primary tumor is higher than in the metastatic lymph nodes. According to the study of Fok et al [23], STK31 is robustly and heterogeneously expressed in colon cancer tissues and plays a critical role in determining the differentiation state of colon cancer cells. Thus, the inconsistency may be related to different tumor differentiation in the primary tumor site and the lymph node metastases, Moreover, the discrepancy might underline the important role of STK1 in the early metastatic processes. Further studies on larger samples to investigate the sensitivity and specificity of STK31 for predicting metastatic CRC risk are needed. Measurements of mRNA and STK31 protein levels in tumor cells with high and low metastatic potential by RT-PCR, immunohistochemistry analysis and flow cytometry are warranted. Moreover, exploration of the prognostic value of STK31expression for metastasis formation and predictive value for overall- and disease-free survival (DFS) after a long-term follow-up is ongoing.

In conclusion, our study is to our knowledge the first attempt to analyze STK31 expression in Chinese CRC population in relation to different clinicopathological features. We found a higher relative STK31 expression level in patients with lymph node metastasis, and demonstrated the diagnostic and prognostic potential of STK31 transcripts in colorectal cancer patients. High STK31 levels in the primary tumor may be prognostic for high risk of metastasis and correlate with an increased patient mortality, rendering it a potential prognostic factor, biological marker of tumor invasiveness and therapeutic target.

## MATERIALS AND METHODS

### Patients and tissue samples

A total of 44 CRC patients who were treated at Shanghai East Hospital from October 2009 until April 2010 were enrolled in the study. Surgical specimens of cancerous tissue, paired adjacent noncancerous tissue and lymph node were obtained from all cases, in accordance with the Ethics Committee of Shanghai East Hospital after obtaining an informed consent. Patients’ data on age, tumor sites, multiplicity, histologic grade, invasion depth, presence of lymph node metastasis, necrosis, lymphatic and the number stage stages were collected. The sample included 27 men and 17 women, with a mean age of 67 years (median 67 years; in women 63 years; in men 68 years; range 43–91 years). Histological tumor staging was performed by routine pathology. The detailed clinicopathological characteristics are summarized in Table 2. All tissue samples were incubated in RNA Later (Ambion, Austin, Texas, USA) at 4°C for 24 hours and stored at −80°C until RNA extraction.

**Table 2:** Clinicopathological characteristics of the study patients

Characteristics	Specifics	No. of metastatic CRC patients (20)	No. of non-metastatic CRC patients (24)
**Age distribution**	≤ 60 y	14 (70.0%)	9 (37.5%)
	> 60 y	6 (30.0%)	15 (62.5%)
**Gender**	Male	14 (70.0%)	13 (54.2%)
	Female	6 (30.0%)	11 (45.8%)
**CRC location**	Colon	8 (40.0%)	8 (33.3%)
	Rectum	12 (60.0%)	16 (66.7%)
**Histologic grades**	Well or moderately differentiated	15 (75.0%)	20 (83.3%)
	Poorly differentiated	5 (25.0%)	4 (16.7%)
**T stage**	T2	3 (15.0%)	2 (8.3%)
	T3	12(60%)	17(70.8%)
	T3, T4	5 (25.0%)	5 (20.8%)
**Stage at diagnosis**	Stage I + II	12 (60.0%)	13 (54.2%)
	Stage III + IV	8 (40.0%)	11 (45.8%)

### Measurement of STK31 expression

#### Total RNA extraction and reverse transcription

Total RNA isolated from tumor, adjacent normal tissue and lymph node samples was prepared with Trizol Reagent (Invitrogen, Carlsbad, CA, USA), according to manufacturer's protocol. Reverse transcription was performed with 1 μg total RNA and MMLV reverse transcriptase (Promega, Madison, Wisconsin, USA). cDNA was stored at −80°C.

### Quantitative real-time polymerase chain reaction (QRT-PCR)

Reactions were performed using Syber green PCR Master Mix (Takara Biotech, Dalian, China) in real-time PCR apparatus (iCycler iQ5, Bio-Rad, Hercules, CA, USA). Sense primer 5′-AAATCTGTGAGTCAGCGAGCCT-3′ and antisense primer 5′-TGATCCACTTTGGGGATTCC AT-3′ were used to amplify STK31 gene; glyceraldehyde-3-phosphate dehydrogenase (GAPDH; sense primer 5′-AGAAGGCTGGGGCTCATTTG-3′ and antisense primer 5′- AGGGGCCATCCACAGTCTTC -3′) as internal control. Primers were synthesized by Sangon Biotech (Shanghai) Co., Ltd. (China). The QRT-PCR was performed in strict accordance with the kit instructions. Thermal cycling for all reactions was initiated with a denaturation step at 95°C for 10 min, followed by 40 cycles at 95°C for 5 s and 60°C for 45 s. Each assay was performed as triplicate. Results were quantitated using comparative cycle threshold (CT) method 2^−ΔCT^ (You et al. 2010). All calculated concentrations of the target gene were divided by endogenous reference (GAPDH) to obtain normalized STK31 expression values.

### Immunohistochemistry

In brief, formalin-fixed, paraffin-embedded tissue sections were dewaxed and the antigens retrieved. The primary antibody against STK31 (abcam, USA) was diluted 1:40. Secondary anti-mouse antibodies were applied on slides for 1 h at 37°C, followed by diaminobenzidine and hematoxylin treatment. PBS was used in the same manner as a substitute for the primary antibody and used as negative control.

### Statistical analysis

Statistical calculations were performed using SPSS version 17.0 (SPSS, Chicago, IL). Differences between groups were estimated using Student's *t* test (Independent-Samples *t* test or Paired-Samples *t* test) and One-way ANOVA analysis. A two-sided significance level of 0.05 (confidence level > 95%) was used for all statistical tests.
